# Carnosic acid prevents dextran sulfate sodium-induced acute colitis associated with the regulation of the Keap1/Nrf2 pathway

**DOI:** 10.1038/s41598-017-11408-5

**Published:** 2017-09-08

**Authors:** Neng Yang, Zongling Xia, Naiyuan Shao, Bowen Li, Lian Xue, Ya Peng, Feng Zhi, Yilin Yang

**Affiliations:** 1 Office of Drug Clinical Trial Institution, The First People’s Hospital of Changzhou, Changzhou, Jiangsu China; 2 Department of Pharmacy, The First People’s Hospital of Changzhou, Changzhou, Jiangsu China; 3 Department of Neurosurgery, The First People’s Hospital of Changzhou, Changzhou, Jiangsu China; 4 Modern Medical Research Center, The Third Affiliated Hospital of Soochow University, Changzhou, Jiangsu China

## Abstract

Crohn’s disease and ulcerative colitis are inflammatory bowel diseases (IBDs) with high prevalence in humans. Carnosic acid (CA) has been reported to possess antioxidative properties; however, its role in IBDs has not been determined. In the present study, we found that CA significantly prevented the loss of body weight and shortening of colon length in acute colitis induced by dextran sodium sulfate (DSS). Pronounced infiltration of immune cells and a loss of crypt architecture and goblet cells were ameliorated by CA. CA significantly decreased the activity of MPO and infiltration of F4/80^+^ macrophages in the colon. DSS-induced pro-inflammatory cytokine mRNA and protein levels in the colon were also attenuated by CA. CA decreased the activation of p65 and c-Jun signalling. CA inhibited DSS-induced NLRP3 inflammasome activation by reducing caspase 1 activity. In addition, CA increased the level of Nrf2 and prevented the degradation of Nrf2 via ubiquitination by blocking the interaction between Cullin3 and Keap1, which resulted in the decrease of Nrf2 target genes. Finally, GSH levels and SOD activity were increased after CA treatment, while MDA and iNOS levels were significantly reduced. Taken together, our data showed that CA may be useful as a potential therapeutic candidate for IBDs.

## Introduction

Crohn’s disease (CD) and ulcerative colitis (UC) are chronic, progressive immunologically mediated diseases that affect millions of people worldwide^1^. It has been reported that the incidences of CD and UC are the highest in northern Europe, the United Kingdom, and North America^2^. Chronic inflammation of the colonic mucosa leads to the reduction in quality of life for CD and UC patients, and it is associated with abdominal pain, diarrhoea, bloody stool and weight loss^3^. Treatment for UC mainly consists of sulfasalazine, corticosteroids, immunosuppressive drugs and monoclonal antibodies against tumour necrosis factor α (TNF-α)^4^. Infliximab, a chimeric monoclonal antibody directed against TNF-α, has been considerably successful in treating CD. However, recent studies showed that infliximab yielded conflicting results in UC patients^5^. Sulfasalazine, a prodrug composed of 5-aminosalicylic acid (5-ASA) and sulfapyridine, has been used in the treatment of inflammatory bowel diseases (IBDs), including CD and UC, as a standard-of-care for decades^6^. The use of sulfasalazine has been associated with a variety of adverse effects, such as blood disorders, hepatotoxicity, ulcerogenic potential, hypospermia and male infertility^7–9^. The pursuit of safer and more effective agents with fewer adverse effects is urgently needed.

It is well established that oxidative stress is a potential pathogenic factor that triggers the IBD inflammation reaction^10^. Inflammatory cells such as neutrophils and macrophages generate large amounts of reactive oxygen species (ROS), which are by-products of the normal metabolism of oxygen. It is well known that many transcription factors, such as NF-κB, AP-1, PPAR-γ, p53, Nrf2 and HIF-1α, can be activated by oxidative stress and regulate the expression of over 500 genes, which encode for growth factors, inflammatory cytokines, or chemokines or related to the cell cycle and cellular regeneration^11^. Nrf2, a key regulator of various genes encoding phase II detoxifying enzymes and antioxidant genes, regulates the basal and inducible expression of numerous detoxifying and antioxidant proteins^12^. On the other hand, numerous studies have reported that the generation of ROS is required for the activation of JNK and NF-κB, which are involved in inducing inflammatory cytokines^13^.

Rosemary (*Rosmarinus officinalis*, Lamiaceae) has been used in the food industry as a flavouring agent and to provide a major source of natural antioxidants^14^. Carnosic acid (CA), a naturally polyphenolic diterpene derived from the rosemary plant, has a wide array of pharmacological and biological activities. An increasing amount of evidence has shown that as an antioxidant, CA protects against oxidative stress-induced cytotoxicity and tissue injury^15, 16^. CA induces apoptosis through the ROS-mediated endoplasmic reticulum stress response^17^. CA modulates CHOP/GADD153 to promote androgen receptor degradation and decreases xenograft tumour growth^18^. Moreover, it has been reported that CA exhibits an antioxidative property by binding to specific Keap1 cysteine residues, which in turn activates the Keap1/Nrf2 pathway^19^. As an Nrf2-ARE activator, CA has been shown to decrease mitochondrial dysfunction, oxidative damage and neuronal cytoskeletal degradation following traumatic brain injury in mice^20^. Although a recent study reported that CA suppresses colon tumour formation in association with antiadipogenic activity^21^, the effect of CA on colitis has not been investigated. In the present study, we evaluated the protective roles of CA in dextran sodium sulfate (DSS)-induced experimental colitis. We found that CA prevented DSS-induced colitis by regulating the Keap1/Nrf2 pathway, ameliorating oxidative stress and inhibiting pro-inflammatory cytokine production.

## Results

### Carnosic acid prevented DSS-induced acute colitis in mice

**Figure 1:**
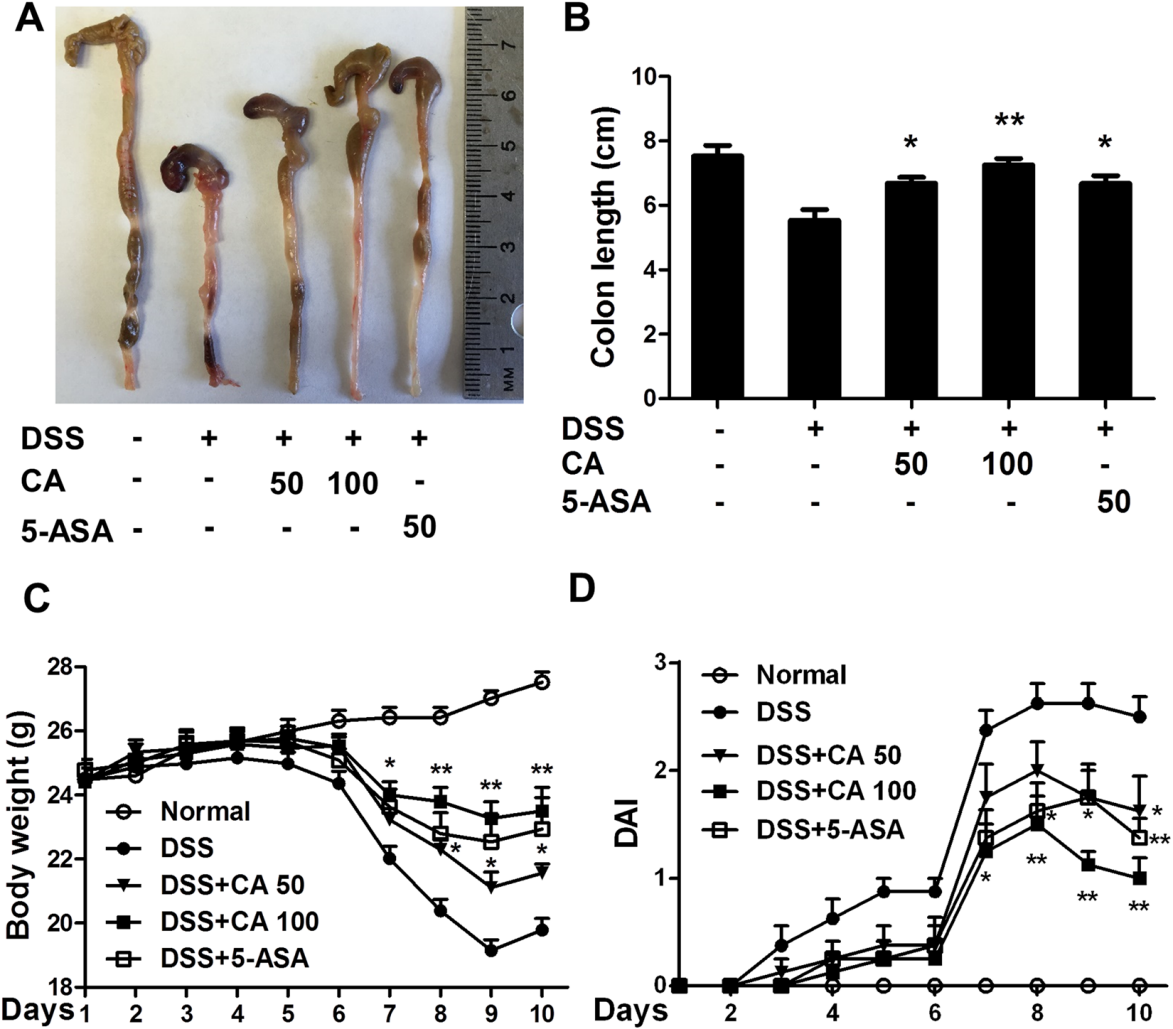
Carnosic acid prevented DSS-induced murine experimental colitis. Mice were treated with 3% DSS drinking water for 7 days, followed by normal water for 3 days. Vehicle, CA (50 and 100 mg/kg) or 5-ASA (50 mg/kg) was administered by gavage once daily from day 1 to day 10. Macroscopic appearances (**A**), colon length (**B**), body weight (**C**) and disease activity index (**D**) were measured and calculated. The data are shown as the means ± SEM, n = 8. *P < 0.05, **P < 0.01 vs. DSS-treated group.

To evaluate the protective effect of CA on DSS-induced murine experimental colitis, mice were exposed to 3% DSS in their drinking water for 7 days, followed by normal water for 3 days. As shown in Fig. 1A, DSS typically induced colonic shortening, gross bleeding and watery feces. Although morphological changes were not completely alleviated after CA or 5-ASA treatment compared to normal mice, Fig. 1B showed that CA at 50 and 100 mg/kg and 5-ASA at 50 mg/kg significantly limited DSS-induced colonic shortening in a dose-dependent manner. Moreover, it was found that gross bleeding in the stool as well as body weight loss were early symptoms that occurred around day 6 after DSS administration. CA or 5-ASA treatment limited DSS-induced body weight loss (Fig. 1C). DAI score, a clinical parameter reflecting rectal bleeding and stool consistency, was dramatically increased at day 7. Compared to the vehicle treatment, CA treatment significantly decreased DAI score from day 7 to 10. These data suggest that CA prevented DSS-induced acute colitis in mice.

### Carnosic acid inhibited F4/80^+^ macrophage infiltration in DSS-induced colitis

**Figure 2:**
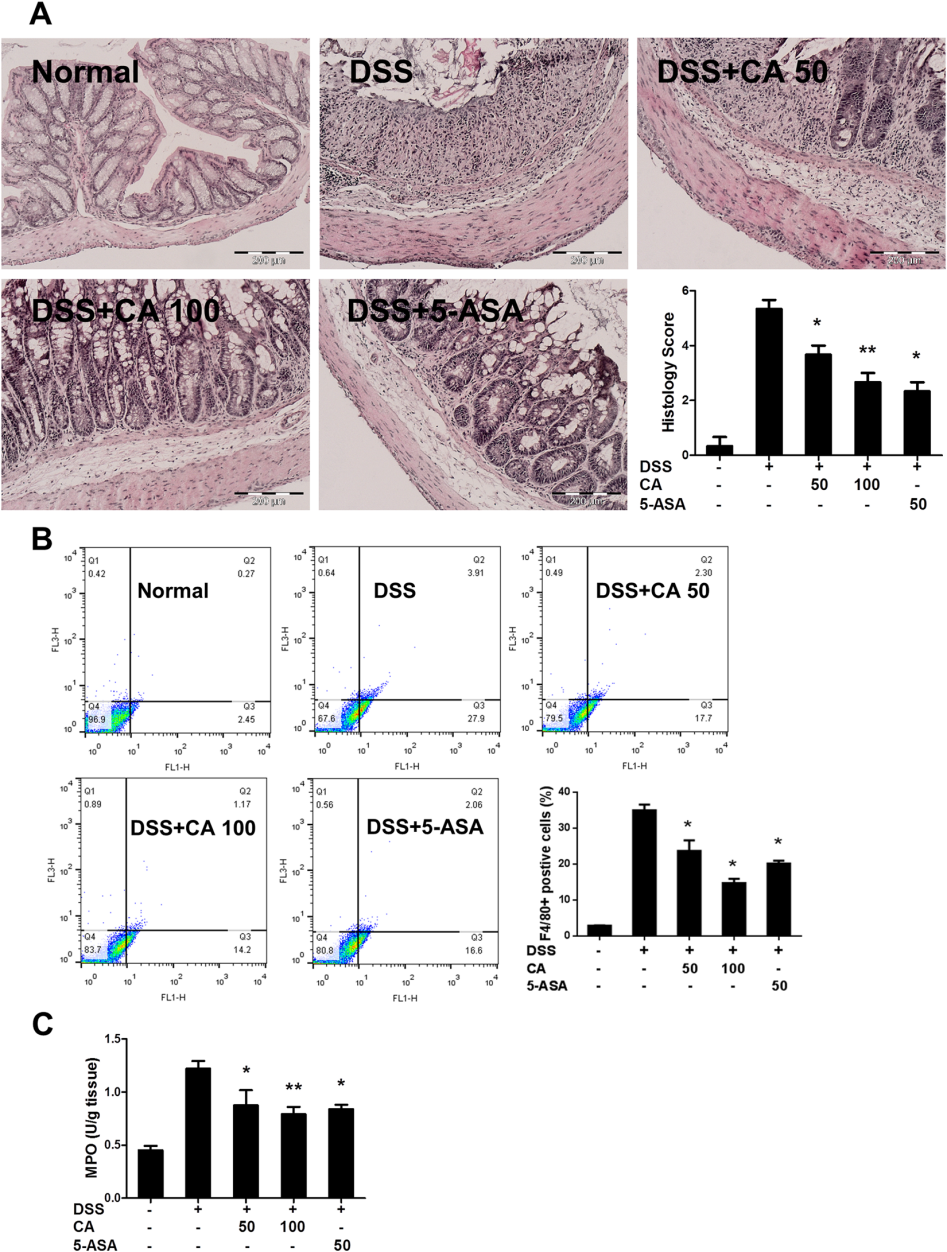
Carnosic acid inhibited macrophage infiltration in DSS-induced colitis. (**A**) Representative H&E stained colon sections of healthy mice with colitis treated with and without carnosic acid. Magnification: 200×. Histopathological scores of each group were determined. (**B**) Percentage of F4/80+cells in the infiltrated leukocytes of colon tissue was determined by flow cytometry. (**C**) Colonic tissue was homogenized, and the levels of MPO were measured using commercial kits. The data are shown as the means ± SEM, n = 8 *P < 0.05, **P < 0.01 vs. DSS-treated group.

We further confirmed the effects of CA on DSS-induced colitis using histopathological analyses. Tissues from mice that did not receive DSS treatment showed intact surface epithelium, intact mucosa and submucosa, non-disrupted crypt architecture, and complete goblet cells with mucus vacuoles. Tissues from mice treated with DSS showed crypt distortion, goblet cell loss, pronounced inflammatory cell infiltration into the submucosa, and mucosa thickening with abundant oedema in the colon. CA at 50 and 100 mg/kg or 5-ASA at 50 mg/kg alleviated these pathological changes with less crypt destruction and goblet cell loss. The histology scores of CA-treated mice were significantly decreased in a dose-dependent manner when compared with the vehicle group (Fig. 2A). Macrophage infiltration plays a key role in acute colitis. Therefore, we investigated whether CA reduced the number of F4/80^+^ macrophages in the colonic mucosa. As shown in Fig. 2B, the percentage of infiltrating macrophages (F4/80^+^) was increased after DSS challenge, and CA or 5-ASA treatment reduced the percentage of F4/80^+^ cells in the colon. Next, we investigated the effect of CA on the activity of myeloperoxidase, which is related to neutrophil, eosinophil and macrophage infiltration. In line with the flow cytometry results, CA treatment reduced the DSS-induced increase in MPO activity (Fig. 2C), indicating that CA inhibited macrophage infiltration in the DSS-treated mice.

### Carnosic acid reduced DSS-induced pro-inflammatory cytokine production in the colon

**Figure 3:**
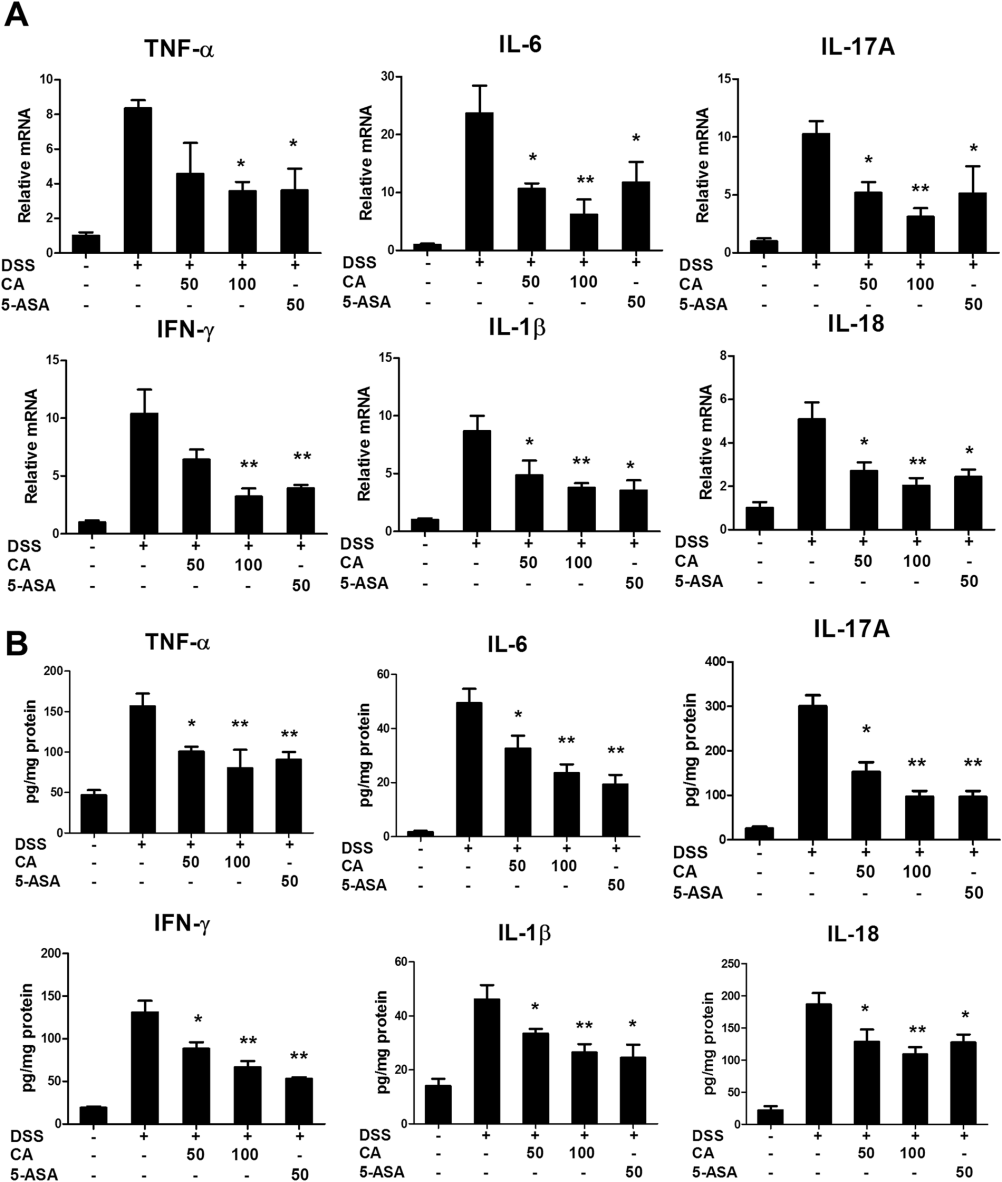
Carnosic acid inhibited DSS-induced inflammatory cytokine expression in the colon. (**A**) The mRNA expression levels of the inflammatory genes TNF-α, IL-17A, IL-6, IFN-γ, IL-1β and IL-18 in colonic tissues were determined by real-time PCR. (**B**) The levels of inflammatory proteins TNF-α, IL-17A, IL-6, IFN-γ, IL-1β and IL-18 in colonic tissues were determined by ELISA. The data are shown as the means ± SEM, n = 8 *P < 0.05, **P < 0.01 vs. DSS-treated group.

It is well known that Crohn’s disease and ulcerative colitis are characterized by the production of a wide range of inflammatory cytokines^22^. To determine the effect of CA on cytokine production in mice with DSS-induced colitis, both mRNA and protein levels of cytokines expressed in the colon were measured using real-time PCR and ELISA. As shown in Fig. 3A, DSS treatment significantly induced the expression of the pro-inflammatory genes TNF-α, IL-17A, IL-6, IFN-γ, IL-1β and IL-18 in the colon, while CA or 5-ASA treatment markedly reduced the expression of these genes in the colon. Consistent with the real-time PCR results, ELISA results showed that CA treatment inhibited the DSS-induced TNF-α, IL-17A, IL-6, IFN-γ, IL-1β and IL-18 protein levels in the colon (Fig. 3B). These results indicated that CA prevented DSS-induced colitis by inhibiting inflammatory cytokine production.

### Carnosic acid decreased NF-κB and c-Jun signalling in DSS-induced colitis

**Figure 4:**
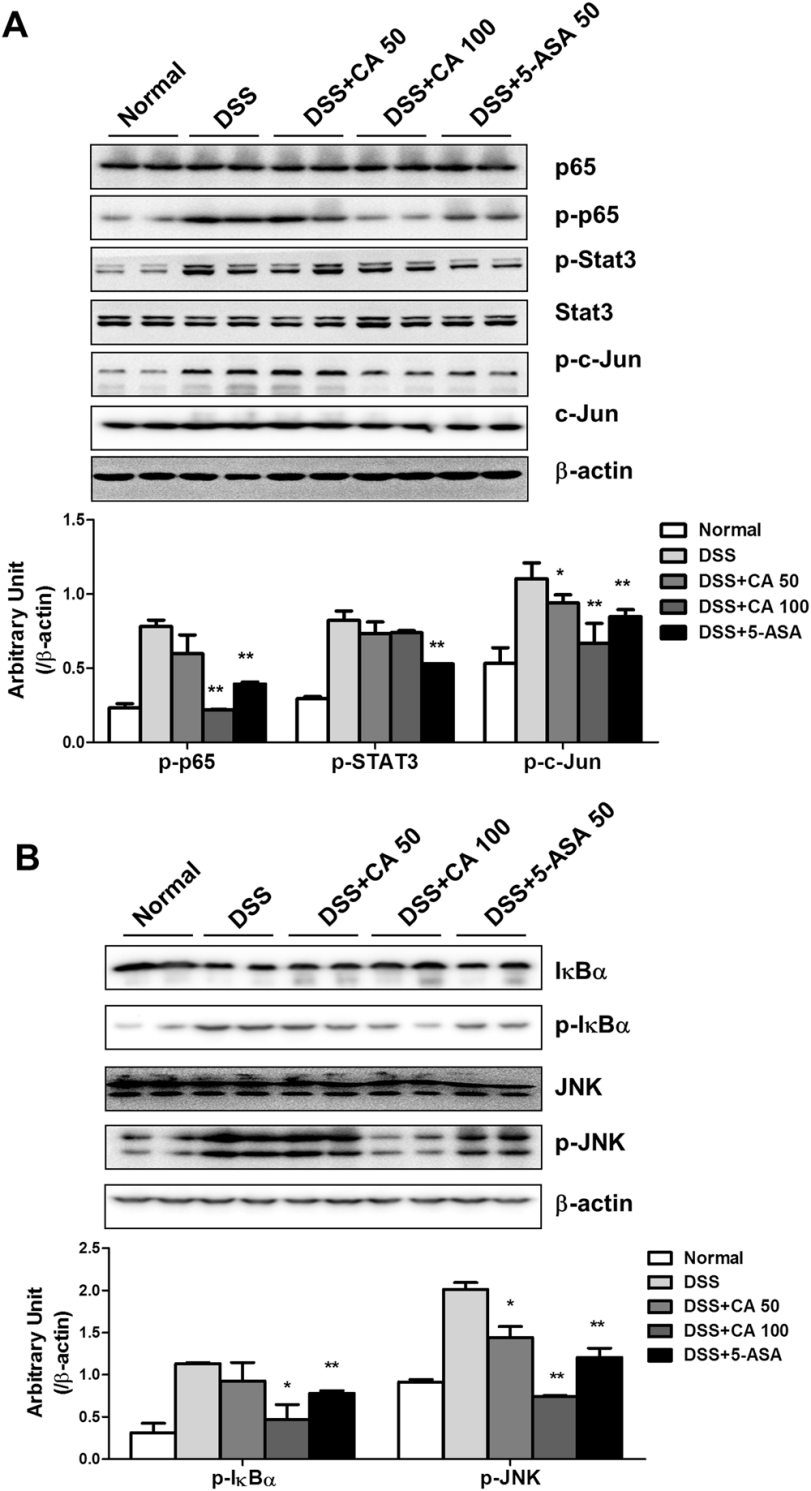
Carnosic acid decreased the activation of NF-κB and c-Jun signalling. (**A**) The levels of total and phosphorylated p65, Stat3, c-Jun, IκBα and SAPK/JNK proteins in the colon were determined by Western blotting. β-Actin was used as the endogenous control. Representative data are shown, and the bands were analysed by densitometry. The data are shown as the means ± SEM. *P < 0.05, **P < 0.01 vs. DSS-treated group.

We further investigated the mechanism behind the protective role of CA in DSS-induced colitis. The roles of transcription factors, including NF-κB, Stat3 and c-Jun, in the regulation of inflammation have been well described^23^. A Western blot assay was performed to determine the effect of CA on the activation of these signalling pathways. As shown in Fig. 4A, DSS significantly increased p65, Stat3 and c-Jun phosphorylation in the colon, indicating that DSS dramatically activated these signalling pathways. Interestingly, CA treatment decreased p65 and c-Jun phosphorylation but not Stat3 phosphorylation. We further assessed the effects of CA on upstream proteins of p65 and c-Jun. CA treatment significantly inhibited IκBα and JNK phosphorylation in the colons of DSS-treated mice (Fig. 4B). These results indicated that CA inhibited macrophage activation by suppressing NF-κB and c-Jun signalling, thereby reducing cytokine production.

### Carnosic acid inhibited DSS-induced NLRP3 inflammasome activation

**Figure 5:**
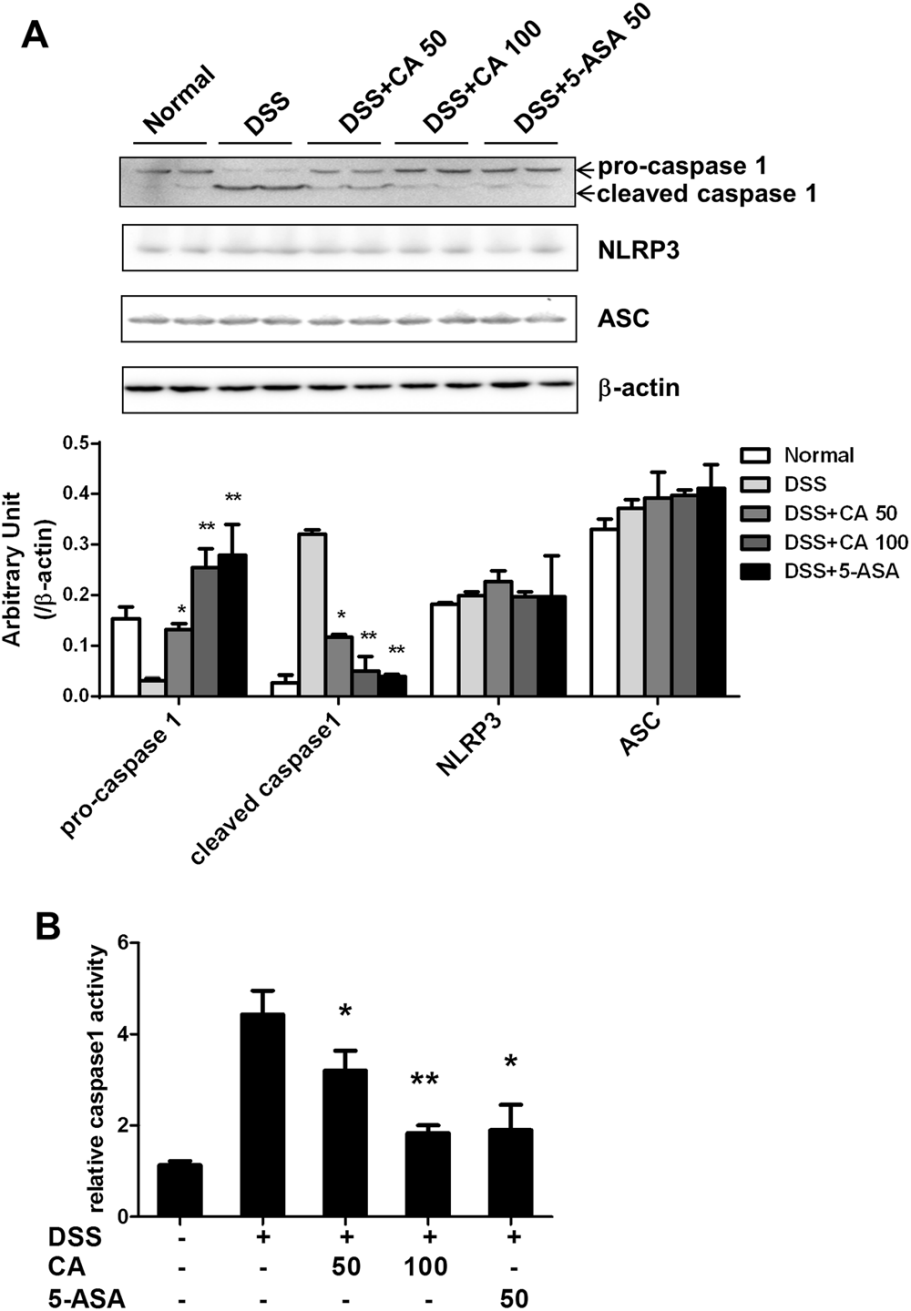
Carnosic acid inhibited DSS-induced NLRP3 inflammasome activation. (**A**) The levels of pro-caspase 1, cleaved caspase 1, NLRP3 and ASC proteins in the colon were determined by Western blotting. β-Actin was used as the endogenous control. Representative data are shown, and the bands were analysed by densitometry. (**B**) The activity of caspase 1 in the colonic tissue was determined using a commercial kit.

An increasing number of studies have shown that the inflammasome is involved in colitis. We further examined the effect of CA on DSS-induced inflammasome activation. As shown in Fig. 5A, the DSS challenge inhibited pro-caspase 1 expression in the colon, whereas CA treatment significantly prevented the decrease in pro-caspase 1 expression and inhibited the increase in cleaved caspase 1 expression, which contributed to NLRP3 inflammasome activation in response to DSS. However, CA or 5-ASA treatment could not alter the levels of NLRP3 and ASC in the colon. In addition, we found that CA inhibited caspase 1 activity in DSS-treated mice. These data indicated that the inhibition of inflammasome activation is involved in the amelioration of experimental murine colitis by CA.

### Carnosic acid prevented the decrease in DSS-induced Nrf2 activation

**Figure 6:**
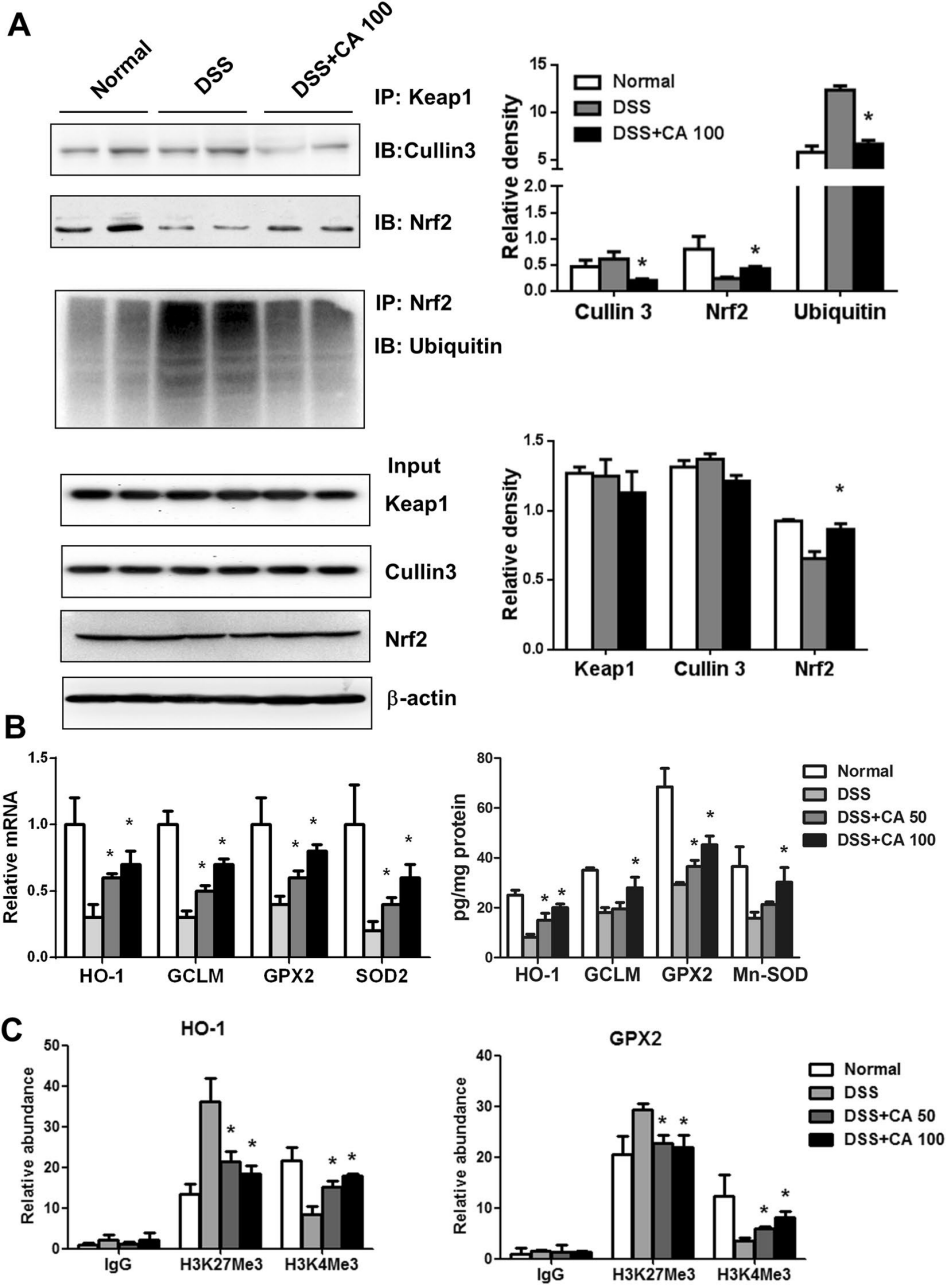
Carnosic acid prevented the decrease in DSS-induced Nrf2 activation. (**A**) Keap1 protein in the colonic tissue was immunoprecipitated with anti-Keap1 antibody or IgG. The interactions between Keap1 and Cullin 3 or Nrf2 were determined by Western blotting. Nrf2 protein in the colonic tissue was immunoprecipitated with anti-Nrf2 antibody, and the level of Nrf2 ubiquitination was determined by Western blotting. (**B**) The mRNA and protein expression of Nrf2 target genes HO-1, GCLM, GPX2 and SOD2 in the colonic tissues was determined by real-time PCR and ELISA assay. (**C**) Fresh frozen sections of colons were homogenized in PBS. Chromatin was cross-linked with 37% formaldehyde, sheared to an size of ~0.5–1 kb and incubated with either anti-H3K27Me3, anti-H3K4Me3, or IgG (negative control). The binding ability of anti-H3K27Me3 and anti-H3K4Me3 to Nrf2 target gene HO-1 or GPX2 promoter was determined by Real-time PCR. The data are shown as the means ± SEM. *P < 0.05, **P < 0.01 vs. DSS-treated group.

Oxidative stress and inflammation are always associated with each other, and the use of antioxidants can be protective against inflammatory diseases^24^. As a key regulator of detoxification, Nrf2 is responsible for the transcriptional activation of genes encoding antioxidant enzymes. Next, we examined whether Nrf2 is responsible for the protective effect of CA in DSS-induced colitis. As shown in Fig. 6A, Co-IP results showed that CA treatment blocked the interaction between Cullin3 and Keap1, which contributes to Nrf2 ubiquitination. We also found that CA prevented the DSS-induced reduction in Nrf2 protein levels. Moreover, Fig. 6A showed that CA inhibited the level of Nrf2 ubiquitination, suggesting that CA may prevent the DSS-induced reduction in Nrf2 protein levels by blocking the interaction between Cullin3 and Keap1, thus contributing to the degradation of Nrf2 via ubiquitination. In line with the Co-IP results, the mRNA expression levels of the Nrf2 target genes HO-1, GCLM, GPX2 and SOD2 in colonic tissues were up-regulated by CA treatment. ELISA assay showed that the protein levels of the Nrf2 target genes in colonic tissues were also up-regulated after CA treatment (Fig. 6B). In addition, we used Chip assay to determine the effect of histone trimethylation on Nrf2 target gene transcription. The data showed that CA treatment increased the binding ability of H3K4Me3 and decreased the binding ability of H3K27Me3 to the promoter of HO-1 and GPX2 repectively (Fig. 6C). This result indicated that CA prevented the DSS-induced reduction in Nrf2 activation.

### Carnosic acid attenuated oxidative stress in DSS-induced colitis

**Figure 7:**
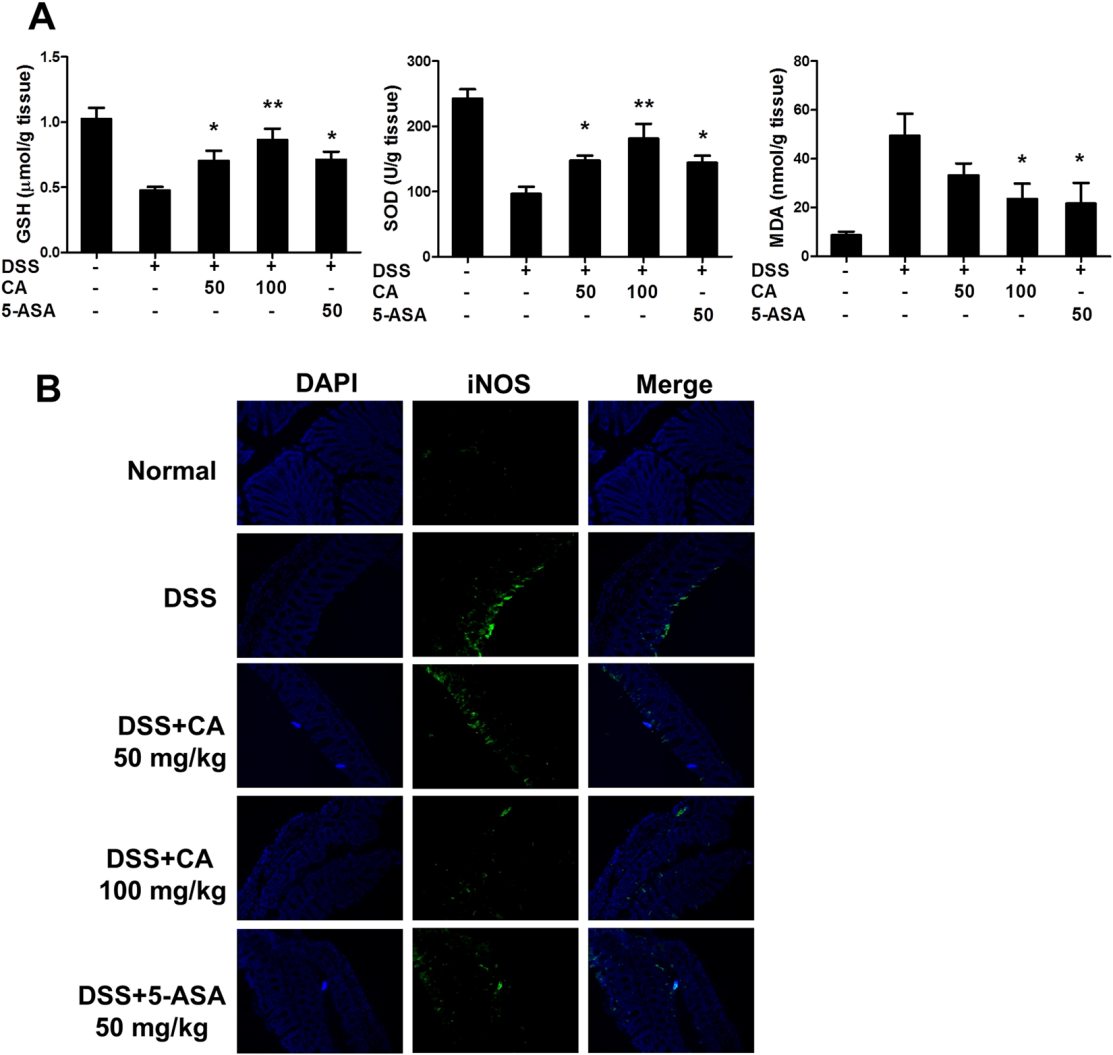
Carnosic acid attenuated oxidative stress in DSS-induced colitis. (**A**) Colonic tissue was homogenized, and the levels of GSH, SOD and MDA were measured using commercial kits. (**B**) Sections of colonic tissue were immunostained with anti-iNOS antibody. The slides were counterstained with DAPI and the images were captured using an inverted fluorescence microscope. Magnification: 200×. The data are shown as the means ± SEM, n = 8 *P < 0.05, **P < 0.01 vs. DSS-treated group.

To confirm the effect of CA on Nrf2 activation, we further examined oxidative stress in DSS-treated mice. As shown in Fig. 7A, the levels of GSH and SOD were decreased after DSS challenge, while CA treatment markedly increased the levels of GSH and SOD in the colon. In contrast, CA at 100 mg/kg or 5-ASA at 50 mg/kg significantly inhibited the DSS-induced elevation of MDA levels. Nitric oxide is a reactive free radical which acts as a biologic mediator in inflammatory diseases such as IBD. Inducible nitric oxide synthase (iNOS) plays a key role in nitric oxide production, inflammation and oxidative stress. We used immunofluorescence histochemistry to determine iNOS expression in colon. The results showed that DSS increased iNOS protein expression in the colons of DSS-treated mice while CA treatment inhibited DSS-induced iNOS upregulation. These results indicated that the attenuation of oxidative stress by CA could play a key role in DSS-induced colitis.

## Discussion

The present study revealed, for the first time, a protective role for CA, the major polyphenolic diterpene of the rosemary plant, in an experimental murine model of Crohn’s disease. We found that CA administration (50 and 100 mg/kg) diminished the severity of intestinal injuries, including loss of body weight and shortening of colonic length. An increasing number of reports have shown that more than 8,000 identified plant polyphenolic compounds constitute one of the largest and most ubiquitous groups of secondary metabolites^25^. Plant polyphenolic extracts and pure polyphenols contribute to a variety of intracellular signalling cascades in intestinal cells to ameliorate inflammatory reactions^26^. Moreover, many compounds from natural products, such as ellagic acid, gallic acid and quercetin, have been shown to prevent IBD through their antioxidative properties^27–29^. Rosemary is widely used and commercialized as an antioxidant in foods, nutritional supplements, and cosmetics. Likewise, CA also exhibits anti-microbial, neuroprotective, hepatoprotective, anti-obesity and anti-cancer properties via attenuating oxidative stress^30^. However, few studies have shown the role of CA in Crohn’s disease and ulcerative colitis. Our data showed that CA significantly increased the levels of GSH and SOD and decreased the level of MDA induced by DSS, suggesting that CA could play a relevant role in the development of therapies for inflammatory diseases associated with oxidative stress.

In patients with CD or UC, oxidative stress is thought to be a major cause of tissue destruction. Reactive oxygen species and nitrogen metabolites play key roles in the initiation and progression of IBDs^31^. Therefore, signalling pathways involved in oxidative stress are investigated as major targets in inflammation research. Our results showed that CA significantly reduced DSS-induced pro-inflammatory cytokine production, macrophage infiltration and MPO activity (Figs 2 and 3). These results suggest that CA may suppress inflammatory cytokine production and macrophage infiltration by attenuating oxidative stress. However, a clinical trial has revealed that the increased production of oxidative free radical by polymorphonuclear cells could be related to the pathogenesis or aggravation of ulcerative colitis^32^. It is likely that CA attenuates oxidative stress through the inhibition of macrophage infiltration and pro-inflammatory cytokine production. Further investigation into the relationship between oxidative stress and inflammation is required to elucidate the mechanism by which CA affects intestinal inflammation.

We evaluated the effects of CA on macrophage functions, and our results showed that CA administration inhibited not only DSS-induced inflammatory cytokine production but also macrophage infiltration. Many transcription factors, such as NF-κB, Stat3 and AP-1, play critical roles in the transcriptional regulation of cytokines and chemokines, such as IL-1β, IL-6 and TNF-α^23^. Our data showed that CA significantly inhibited p65 and c-Jun phosphorylation in DSS-induced colitis. Clearly, CA reduced DSS-induced pro-inflammatory cytokine levels in the colon through the inhibition of p65 and c-Jun activation but not Stat3 activation. Further study showed that CA inhibits DSS-induced phosphorylation of IκBα and JNK, which are important kinases for regulating the activity of transcription factors. In the NF-κB pathway, the IκB kinase (IKK) complex, including two catalytically active kinases (IKKα and IKKβ) and the regulatory subunit IKKγ (NEMO), phosphorylates IκBα, which in turn induces the degradation of IκB proteins^33^. Likewise, numerous studies have reported that many kinases, such as mitogen-activated protein kinase kinase (MKK), phosphorylate stress-activated protein kinase/Jun-amino-terminal kinase SAPK/JNK, which in turn activate c-Jun to regulate gene transcription^34^. However, the roles of CA in DSS-induced IKKα, IKKβ and MKK activation remains unclear. In addition, it has been reported that ROS can induce or mediate the activation of the MAPK and NF-κB pathways^35, 36^. Based on the results of this study, it is likely that both NF-κB and c-Jun signalling are involved in the protective effects of CA on DSS-induced colitis. Importantly, the attenuation of oxidative stress by CA may play a key role in the activation of NF-κB and c-Jun signalling.

In this study, we found that CA treatment prevented the degradation of Nrf2, which in turn up-regulated the transcription of antioxidant genes. Moreover, we found that CA significantly blocked the interaction between Cullin3 and Keap1 (Fig. 6). It is well known that the E3 ubiquitin ligase Cullin3 mediates the degradation of Nrf2^37^. CA treatment reduced the level of ubiquitination of Nrf2 by blocking the interaction between Cullin3 and Keap1. Clearly, Nrf2 activity is regulated by its stability. Therefore, CA may increase Nrf2 activity by increasing its stability. In addition, Nrf2-mediated oxidative stress plays a key role in inducing NLRP3 inflammasome activation^38^. CA may inhibit DSS-induced inflammasome activation through regulating Nrf2-mediated oxidative stress. Although it has been reported that the attenuation of oxidative stress by the activation of Nrf2 is involved in the protective role of CA, the mechanism of action remains unclear. The identification of exact molecular targets of CA is necessary in future studies.

In conclusion, we explored the protective effects of CA in DSS-induced murine experimental colitis. Our results showed that CA exhibited a protective role in DSS-induced experimental colitis through the regulation of the Keap1/Nrf2 pathway. These data strongly suggest that CA may be a promising therapeutic candidate for the treatment of inflammatory bowel disease.

## Materials and Methods

### Reagents

Carnosic acid, 5-aminosalicylic acid (5-ASA), sodium carboxymethyl cellulose, haematoxylin and eosin were purchased from Sigma Aldrich (St. Louis, MO). Dextran sodium sulfate was purchased from MP Biomedicals (Solon, OH). Anti-p65 (#8242), anti-phospho-p65 (#3039), anti-IĸBα (#9242), anti-phospho-IĸBα(#2859), anti-Stat3 (#4904), anti-phospho-Stat3 (#9145), anti-JNK (#9252), anti-phospho-JNK (#4668), anti-c-Jun (#2315), anti-phospho-c-Jun (#2361), anti-NLRP3 (#15101), anti-ASC (#67824), anti-Ubiquitin (#3933), iNOS (#13120), anti-H3k27Me3 (#9733), anti-H3k4Me3 (#9751) and anti-β-actin (#4970) antibodies were purchased from Cell Signaling Technology (Beverly, MA). Anti-Keap1 (ab150654), anti-Nrf2 (ab62352) and Cullin3 (ab75851) antibodies were purchased from ABCAM. Anti-caspase1 (sc-56036) antibody was purchased from Santa Cruz Biotechnology, TRIzol reagent, TaqMan primers and TaqMan PCR Master Mix were purchased from Invitrogen (Carlsbad, CA).

### Mice

Male Balb/c mice (6–8 weeks of age) were purchased from the Experimental Animal Center of Yangzhou University (Jiangsu, China). The mice were given free access to pellet food and water in stainless-steel cages and maintained at a temperature of 21 ± 2 °C with a 12 h light/dark cycle. All the animal experiments were approved by the Soochow University Animal Care and Use Committee (SDU-ACUC). The experiments were carried out in accordance with the relevant guidelines and regulations from SDU-ACUC and were designed to minimize suffering and to reduce the number of animals used. All the animals received humane care in compliance with the Guide for the Care and Use of Laboratory Animals.

### Induction and Evaluation of Colitis

To induce experimental colitis, mice were treated with 3% (wt/vol) dextran sodium sulfate (DSS, 36,000 to 50,000 MW) in drinking water ad libitum for 7 days, followed by normal water for 3 days. The mice treated with DSS were gavaged with vehicle (0.5% sodium carboxymethyl cellulose), CA (50 and 100 mg/kg) or 5-ASA (50 mg/kg) once daily from day 1 to day 10 (n = 8 per group). The doses of CA and 5-ASA were chosen based on previous studies^39, 40^. All mice were anaesthetized by intraperitoneal injection with a mixture of ketamine and xylazine and sacrificed by heart puncture exsanguination. The disease activity index (DAI), including stool consistency and occult blood, were determined daily until day 10 as described previously^41^. In brief, the stool scores were determined as follows: 0 = well-formed pellets; 1 = semi-formed stools that did not adhere to the anus; and 2 = semi-formed stools that adhered to the anus. The bleeding scores were determined as follows: 0 = no blood; 1 = slight bleeding; and 2 = gross bleeding. DAI was calculated by combining the stool score and bleeding score. At day 10 following induction with DSS, mice were sacrificed for the collection of blood and colonic tissue.

### Histopathological Analysis

For the evaluation of colitis, the colonic tissues were washed, fixed in 10% formalin solution and embedded in paraffin. The tissue sections were stained with haematoxylin & eosin (H&E). Histology was scored by a pathologist in a blinded fashion as described previously^41^. In brief, the inflammatory cell infiltration scores were determined as follows: 0 = occasional inflammatory cells in the lamina propria; 1 = increased numbers of inflammatory cells in the lamina propria; 2 = confluence of inflammatory cells extending into the submucosa; and 3 = transmural extension of the infiltrate. The tissue damage scores were determined as follows: 0 = no damage; 1 = lymphoepithelial lesions; 2 = surface mucosal erosion or focal ulceration; and 3 = extensive mucosal damage and extension into deeper structures of the bowel wall. The combined histological score ranged from 0 (no changes) to 6 (extensive infiltration and tissue damage).

### Real-time PCR

Total RNA from the colon samples was isolated using TRIzol Reagent (Invitrogen, Carlsbad, CA) according to the manufacturer’s instructions. Total RNA (2 µg) was reverse transcribed using a RETROscript® reverse transcription kit (Invitrogen, Carlsbad, CA). RT-PCR was performed with an ABI Prism 7500 sequence detection system (Applied Biosystems, Foster City, CA) using TaqMan primers (Thermo Fisher Scientific, MA) and PCR Master Mix. Amplification of GAPDH was used as an internal control. Relative mRNA expression was quantified using the comparative CT (Ct) method and expressed as 2^(−ΔΔCt)^. The primers used were as follows: TNF-α (Mm00443258_m1), IL-17A (Mm00439618_m1), IL-6 (Mm00446190_m1), IFN-γ (Mm01168134_m1), IL-1β (Mm00434228_m1), IL-18 (Mm00434226_m1), HO-1(Mm00516005_m1), GCLM (Mm01324400_m1), GPX2 (Mm00850074_g1) and SOD2 (Mm01313000_m1).

### ELISA

In brief, fresh frozen sections of colons were homogenized and lysed in RIPA buffer containing protease inhibitor cocktails (Cell Signaling Technology, Danvers, MA). Protein quantification was performed using a BCA Protein Assay Kit (Pierce, Rockford, IL) according to the manufacturer’s instructions. The levels of IFN-γ, IL-1β, IL-6 and TNF-α in the colon homogenate were quantified using ELISA kits (R&D System, Minneapolis, MN). The levels of HO-1, GCLM, GPX2 and Mn-SOD in the colon homogenate were quantified using ELISA kits (Life span biosciences, WA).

### Measurement of MPO, GSH, SOD and MDA

The levels of myeloperoxidase (MPO), glutathione (GSH), superoxide dismutase (SOD) and malondialdehyde (MDA) were measured using commercial kits (Nanjing Jiancheng Bioengineering Institute, Nanjing, China). In brief, colonic tissue was homogenized in ice-cold PBS. After centrifugation, the supernatant was used for the measurement of MPO, GSH, SOD and MDA levels according to the manufacturer’s instructions.

### Western Blot

In brief, fresh frozen sections of colons were homogenized and lysed in RIPA buffer containing protease and phosphatase inhibitor cocktails (Cell Signaling Technology, Danvers, MA). Protein quantification was performed using a BCA Protein Assay Kit (Pierce, Rockford, IL) according to the manufacturer’s instructions. The samples were suspended in loading buffer and boiled, and 50 μg of protein was run on an SDS-PAGE gel and subsequently electro-transferred onto a polyvinylidene difluoride membrane (Millipore Corp., Bedford, MA). The membrane was blocked with 5% non-fat milk, incubated with the indicated primary antibodies, and then incubated with horseradish peroxidase-conjugated secondary antibodies (Cell Signaling Technology, Danvers, MA). The immunoreactive bands were visualized with an enhanced chemiluminescence reagent. The level of β-actin was used as an internal control. ImageJ software was used for densitometric analysis.

### Co-immunoprecipitation

Briefly, the colonic tissue was homogenized with lysis buffer containing Triton X-100, and the lysates were immunoprecipitated with antibodies against keap1, Nrf2 or IgG and protein A/G-Sepharose. The antibody-protein A/G-Sepharose complex was washed, and the proteins were separated by SDS-PAGE gel. Protein bands were visualized using the Western blotting detection system. ImageJ software was used for densitometric analysis.

### Flow Cytometry

In brief, colon tissue was cut into small fragments and rinsed with ice-cold PBS. The fragments were digested in Ca^2+^- and Mg^2+^-free Hank’s buffered saline with 5 mM EDTA and shaken at 200 g for 20 min at 37 °C. Then, the fragments were digested in Hank’s buffered saline containing 1 mg/ml collagenase II and 1 mg/ml dispase and shaken at 200 g for 1 h at 37 °C. The obtained single cells were filtered through a 50 μm cell strainer, and the colon-infiltrating leukocytes were layered on a Percoll gradient. After the cells were washed twice with ice-cold PBS, the leukocytes were suspended in 1 ml PBS containing 1% BSA and 0.05% NaN_3_. The cells (1 × 10^6^) were incubated with F4/80-FITC antibody for 30 min and then analysed on a BD Flow Cytometer.

### Caspase 1 Activity Assay

Briefly, fresh frozen sections of colons were homogenized with lysis buffer using a Dounce homogenizer. Then, the supernatant was separated by centrifugation at 10,000 g for 10 min. The activity of caspase-1 was determined by a commercial kit (Abcam, ab39412) according to the manufacturer’s instructions.

### Immunofluorescence histochemistry

For immunofluorescence histochemistry, tissues samples were fixed and embedded using OCT compound. Frozen sections cut into slices 7 μm thick were stained using anti-iNOS antibody. The slides were counterstained with DAPI, and images were captured using inverted fluorescence microscope (Olympus IX71, Tokyo, Japan).

### Chromatin immunoprecipiation assay

Chromatin immunoprecipiation assay was performed by using a commercially kit (Millpore, Bedford, MA) according to the manufacturer’s instruction. In brief, fresh frozen sections of colons were homogenized in PBS. Chromatin was cross-linked with 37% formaldehyde, sheared to an size of ~0.5–1 kb and incubated with either anti-H3K27Me3, anti-H3K4Me3, or IgG (negative control). Real-time PCR was performed to test the binding abilities of anti-H3K27Me3 and anti-H3K4Me3 to Nrf2 target gene promoter. The primers were used according to the previous studies^42, 43^ as follows: mouse HO-1 promoter primer sequence is: forward, AAGAGCTCCACCCCCACCCA; reverse, GGGCTAGCATGCGAAGTGAG; Gpx2 promoter primer sequence is: forward, CATAGATACAATTGGCCCTTCC; reverse, CAGGATGACTTAGCAAAAACAGGT.

### Statistical analysis

The data are expressed as the means ± SEM. Prism software (GraphPad) was used for statistical analyses. One-way ANOVA was used for statistical data analyses. A *p* value less than 0.05 was considered significant.

### Data availability

The datasets generated during and/or analysed during the current study are available from the corresponding author on reasonable request.
